# Assessment and Appraisal of Drug Innovativeness in Italy: Ultimate Evidence on Key Drivers and Consistency

**DOI:** 10.3390/jmahp14020028

**Published:** 2026-05-02

**Authors:** Alvise Verde, Federica Turati, Clara Trimarchi, Carlotta Galeone, Claudio Jommi

**Affiliations:** 1 Sanofi, 20158 Milan, Italy; 2 Department of Clinical Sciences and Community Health, Department of Excellence 2023–2027, University of Milan, 20133 Milan, Italy; 3 Bicocca-Applied Statistics Center (B-ASC), Università degli Studi di Milano-Bicocca, 20126 Milan, Italy; 4 Department of Pharmaceutical Sciences, Università del Piemonte Orientale, 28100 Novara, Italy

**Keywords:** medicines, innovativeness, appraisal, Italy

## Abstract

This study aims to update and integrate empirical evidence on the key drivers and consistency of the appraisals of drug innovativeness in Italy by the Italian Medicines Agency (AIFA), and discuss if this evidence is supportive of the reform and requirements implemented in 2025. Appraisals from July 2017 to December 2024 were retrieved from the AIFA website. The association between the innovativeness appraisal, the innovativeness domains (unmet need/added therapeutic value/quality of evidence) and disease/drug/evidence-specific variables was assessed using odds ratios (ORs) from binary/multinomial logistic regression models. Innovativeness status was strongly associated with added therapeutic value (OR > 70). Medicines for rare diseases were more likely to receive conditional innovativeness (OR = 2.95). Full innovativeness was more frequently recognized for indications including paediatric patients (OR = 3.60). References to severe diseases and patient-reported outcomes (PROs) had a higher, not statistically significant, likelihood of innovativeness, whereas reference to indirect treatment comparisons had a lower likelihood (OR = 0.18). The appraisal process showed high internal consistency, but its regulation needs more specific guidance. The innovativeness regulation was reformed in July 2025, including specific recommendations on the criteria to identify the alternative treatments; the role and robustness of indirect comparisons; and the role and requirements for PROs. Our evidence provides an empirical rationale for this reform.

## 1. Introduction

The approval of a new drug is based on its absolute risk–benefit profile, whereas the assessment and appraisal for price and reimbursement (P&R) require a comparative assessment [1], including the level of the unmet need (existence and validity of alternative treatments) and the added (therapeutic) value.

In the Italian National Health Service (SSN—Servizio Sanitario Nazionale) this comparative assessment is carried out by the Italian Medicines Agency (AIFA—Agenzia Italiana del Farmaco), starting from a dossier submitted by the pharmaceutical companies [2]. This assessment supports (i) the negotiation of P&R, and (ii) the appraisal of innovativeness status, introduced in 2017. Innovative medicines are granted prioritized access, consisting of an immediate inclusion in regional formularies, exemption from mandatory discounts, exemption from clawback and access to a dedicated fund [3]. Immediate access to regional formularies and exemption from clawback are essential, since for non-innovative medicines this access has experienced significant and varied delays across regions, creating disparities in patient access [4]. Exemption from mandatory discounts is important for pharmaceutical companies’ profitability. The innovative fund allows regions to draw from a fund which is separate from the spending cap on ordinary medicines. This spending cap is systematically overcome, and regions are accountable for 50% of the deficit coverage [5].

Innovativeness status can be given only to drugs indicated for serious diseases (life-threatening diseases; diseases producing frequent hospitalizations or causing disabilities that can seriously compromise quality of life). The innovativeness is appraised on the grounds of the unmet need, the added therapeutic value and the quality of the evidence [6]. The unmet need and the added therapeutic value are ranked in five levels. The quality of evidence is ranked in four levels and driven by the Grading of Recommendations Assessment, Development and Evaluation (GRADE) method [7]. The innovativeness is appraised for a single indication and the appraisal model represents a common framework for all diseases. Only rare indications are allowed for a lower GRADE ranking. The 2017 innovativeness regulation does not give explicit requirements for comparative assessment, including the robustness of the evidence from indirect treatment comparisons (ITCs) and the role of Patient-Reported Outcome Measures (PROMs). The appraisals may award full or conditional innovativeness, which provides either all benefits or only immediate access to regional markets, respectively. Full innovativeness has a maximum 36 months validity, whereas conditional innovativeness lasts 18 months. Conditional innovativeness is awarded if the evidence is promising, but not sufficiently mature. Conditional innovativeness could be reappraised and possibly converted into full innovativeness.

The innovativeness appraisal documents are published on the AIFA’s website [8] once an agreement on P&R is reached and published in the Official Gazette. These documents include the innovativeness status awarded, the grade assigned to each domain, and a report with the rationale for the decision taken.

The empirical evidence on the innovativeness appraisals highlighted that the added therapeutic value is the major driver [3,9], the role of the quality of evidence is more uncertain and rare indications play a minor role. Another finding is the internal consistency of the appraisals: e.g., the year of assessment does not play any role. However, this evidence is not updated and has never incorporated other domains that may have an impact, i.e., whether the appraisal has explicitly mentioned the disease severity, the existence and validity of an ITC, or the existence and role played by PROMs.

The innovativeness regulation was changed in 2025 [10]. Innovativeness is now awarded only to medium- and low-prevalence (and severe) diseases. The three criteria (unmet need, added therapeutic value, quality of evidence) have been confirmed, but with specific recommendations on the identification of the alternative treatments and the role and robustness of ITCs, PROMs and PREMs (Patient-Reported Experience Measures). The innovativeness appraisal will classify drugs as “innovative/not-innovative”; “conditional” can no longer be attributed.

This study aims to provide an updated analysis of the drivers of innovativeness status and the appraisals’ consistency. Furthermore, it aims to integrate the previous analyses with other domains: disease severity, ITCs and PROMs being explicitly mentioned in the appraisal documents.

## 2. Materials and Methods

This is a retrospective observational study based on public reports on the innovativeness of medicines published by AIFA, starting from the implementation of Determination No. 1535/2017, covering the period from July 2017 to December 2024 [8]. Each record corresponded to a single AIFA appraisal, referring to a specific drug and its approved therapeutic indication, which represents the unit of analysis of the study. These appraisal reports have been previously used as data sources in similar analyses of innovativeness assessments [3,9].

Key information was manually extracted from each report and organized into a structured Excel spreadsheet. The following data were coded:

- Identifiers (drug-level data): Trade name, active substance (or combination), type (biological vs. synthetic), therapeutic indication, year of publication of the P&R decision in the Official Gazette.
- Explanatory variables (disease-related): Rarity of the disease (rare vs. non-rare) and orphan designation, verified through institutional sources; target population (adults, paediatric, mixed); disease severity according to AIFA criteria.
- Explanatory variables (study design-related): Number of clinical studies considered; mention of PROMs, specifying type (generic vs. specific); use of ITCs; clinical endpoints assessed (overall survival, OS; progression-free survival; or disease-specific endpoints).
- Explanatory and dependent variables (AIFA evaluation): Appraisal of the three AIFA domains (unmet need, added therapeutic value, quality of evidence).
- Dependent variable (AIFA evaluation): Final innovativeness status (non-innovative, conditional innovativeness, full innovativeness).

Further details on the dataset structure, variables, and data extraction process are provided in the Electronic Supplementary Material (ESM).

Categorical data were summarized as counts (*n*) and percentages (%). Associations were tested using chi-square or Fisher’s exact tests, as appropriate. To further quantify the associations, we used models from the logistic regression family according to the nature of the outcomes considered. In particular, we estimated the odds ratios (ORs) of innovativeness vs. non-innovativeness (binary outcome), with the corresponding 95% confidence intervals (CIs), through binary logistic regression models. Additionally, multinomial logistic regression models were applied to estimate the ORs for full and conditional innovative status, each compared with non-innovative status (categorical outcome). Both univariable and multivariable models were fitted. Covariates included in multivariable models were selected based on unadjusted analyses and the relevant literature, and comprised: evaluation period (2017–2019, 2020–2022, 2023–2024), disease rarity, severity, target population, PRO reporting, ITCs mentioned in the appraisal document, and, in turn, the three AIFA domains. Although orphan drug designation was associated with innovativeness status in the unadjusted analysis, it was excluded from the multivariable model due to the high collinearity with disease rarity. To estimate its adjusted effect, we fitted an alternative model excluding the disease rarity variable.

We also investigated the association between drug- and disease-related characteristics and the ratings assigned in the three domains (unmet need and added therapeutic value: maximum, important, moderate, low; quality of evidence: high, moderate, low, very low; ordered variables). Associations were assessed using chi-square/Fisher’s exact tests and by univariable and multivariable ordinal logistic regression models.

A two-sided *p*-value < 0.05 was considered statistically significant. Data analyses were conducted using SAS version 9.4 statistical software (SAS Institute, Inc., Cary, NC, USA).

## 3. Results

A total of 264 documents were initially retrieved (list of medicines and appraisals by indication), reduced to 259 evaluations after removing duplicates (multiple associations of active substances or multi-indication documents). For methodological consistency, four reappraisal reports were excluded, resulting in 255 appraisals included in the final analysis.

**Figure 1:**
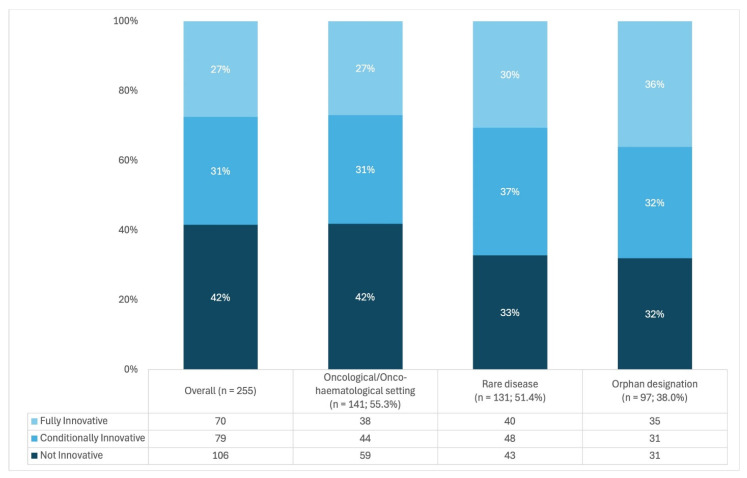
Innovativeness appraisals (*n* = 255).

Of these, 70 (27.5%) were recognized as fully innovative, 79 (31.0%) as conditionally innovative, and 106 (41.6%) as not innovative (Figure 1).

Approximately half of the appraisals (51%) concerned drugs targeting rare diseases, 38% involved drugs with an orphan designation, 55% referred to cancer medicines, 37% to drugs for lethal or life-threatening conditions, 57% to biologic products, and 24% to drugs targeting paediatric-only or mixed populations. Over two-thirds of appraisals (78%) were based on a single pivotal study; PROs were included in 33% of appraisals, ITCs in 24%, and OS results in 51% (Table 1).

Innovativeness was more frequently recognized for drugs targeting rare diseases (*p* = 0.013), those with an orphan drug designation (*p* = 0.021), and those targeting paediatric-only or mixed populations (*p* < 0.001) compared with their counterparts. Innovativeness was less frequently granted when ITCs were mentioned in the appraisal document (Table 1).

The large majority of appraisals received a moderate (58%) or important (33%) rating in the unmet therapeutic need domain (Table 2). In the added therapeutic value domain, 49% of the appraisals were rated as moderate, followed by important (25%) and small (20%) ratings. In the quality of evidence domain, approximately half of the appraisals (47%) received a moderate rating; 13% were rated as high, 28% as low and 12% as very low.

Ratings across the three domains were significantly associated with the final appraisal (Table 2). When increasing numeric scores were assigned to decreasing ratings, the median scores (25–75° percentiles) in the unmet therapeutic need domain were 2 (2–3) for full innovative, 2 (2–3) for conditional innovative, and 3 (2–3) for not innovative appraisals (*p* < 0.001). The corresponding median scores for the added therapeutic value domain were 2 (2–2), 3 (3–3), and 4 (3–4) (*p* < 0.001), and for the quality of evidence domain were 2 (2–3), 2 (2–3), and 3 (2–3) (*p* < 0.001).

The adjusted ORs for innovativeness appraisal are provided in Table 3; unadjusted ORs for all evaluated variables are given in Table S1 of the ESM.

In multivariable analyses that account simultaneously for multiple factors, drugs targeting rare diseases had a higher likelihood of being recognized as innovative (OR 2.59, 95% CI: 1.41–4.78), particularly as conditionally innovative (OR 2.95, 95% CI: 1.48–5.92) (Table 3). Drugs targeting populations including paediatric patients were also more likely to be recognized as innovative (OR 2.19, 95% CI: 0.98–4.45), especially fully innovative (OR 3.60, 95% CI: 1.53–8.44). Similar results were found for drugs with an orphan designation. Appraisals mentioning PROs showed a non-significant trend toward higher likelihood of innovativeness recognition (OR 1.69, 95% CI: 0.88–3.23). Conversely, appraisals reporting ITCs results were less likely to be recognized as innovative (OR 0.18, 95% CI: 0.09–0.36). No significant associations were observed with the oncological setting, biologic drug status, number of supporting studies, or reporting of OS results.

Among the three domains, the strongest association with the final innovativeness appraisal emerged for the added therapeutic value domain (Table 3). The OR for each one-level improvement in the evaluation scale exceeded 70 for the overall innovativeness status and approached infinity and 50 for full and conditional innovativeness statuses, respectively. For the quality of evidence domain, the corresponding ORs were 2.67 for the overall innovative status, 3.79 for the fully innovative status, and 2.12 for the conditionally innovative status. The corresponding figures for the unmet need domain were 1.59, 1.89 and 1.34, respectively.

Results from the analyses assessing the association between drug- and disease-related characteristics and the ratings across the three domains are reported in Tables S2–S4 of ESM. Higher ratings in the unmet need domain were associated with drugs targeting paediatric/mixed populations and rare diseases (non-significant), whereas oncologic indications, the mentioning of ITCs and OS results were associated with lower ratings (Table S2, ESM). In the added therapeutic value domain, higher ratings were associated with drugs targeting paediatric/mixed populations, targeting rare diseases and with oncologic indications (all non-significant); ITCs being mentioned was associated with lower evaluations (Table S3, ESM). For the quality of evidence domain, higher ratings were associated with PROs being mentioned and oncologic indications (non-significant), while lower ratings were associated with ITCs being mentioned, the presence of ≥1 pivotal study, and paediatric/mixed and rare disease target (Table S4, ESM).

## 4. Discussion

This study provides a complete and up-to-date overview of the Italian appraisal of innovativeness. It investigates the determinants of innovativeness status in 255 appraisals from 2017 and 2024, i.e., before the new innovativeness regulation was approved. The analysis has not only extended the period of coverage of previous studies, but has also investigated the role of other variables, e.g., disease severity and ITC and PROM mentions in the appraisal documents.

The analysis confirms the strong association between the innovativeness status and the added therapeutic value, with an OR approaching infinity and 50 for full and conditional innovativeness statuses, respectively. The association with the quality of the evidence, and, in particular, with the unmet need domain, was much lower. A possible explanation for the relatively weak association of the unmet need domain relates to the context in which innovativeness is assessed in Italy. The appraisal is not mandatory and is typically initiated upon request by pharmaceutical companies; it is likely that indications with a relevant unmet need are preferentially selected for application. This may limit the variability of this domain within the analysed sample and possibly explain its comparatively weaker contribution to the final appraisal.

On the other hand, when the associations between the three domains’ ratings and the other variables were investigated, the domain “added therapeutic value” shows less significant association, unlike unmet need and GRADE (Tables S2–S4). This is possibly motivated by the circumstance that, unlike other countries (namely, France and Germany), the quality of the evidence is appraised separately from the added value, making the latter more subject to subjective interpretations [11].

Rare indications were more likely to get innovativeness status. This finding is consistent with the innovativeness regulation, which also allows rare indications to receive the innovativeness with a low quality of evidence, and some of the previous literature findings [3,9]. Conditional approval is more likely than a full one. This result was expected, since the clinical evidence on drugs for rare diseases is less mature at market entry launch [12].

The full innovativeness status was more frequent for indications that included paediatric patients. This result could be attributed to the urgency of making drugs accessible in the paediatric setting.

Appraisals mentioning PROs had a higher, but not statistically significant, likelihood of getting the innovativeness status. The same finding was highlighted for disease severity. Both domains were never considered in the previous literature. One paper showed that if PROs are mentioned in the European Public Assessment Report, there is a higher likelihood of receiving innovativeness status in Italy [13]. Meanwhile, the authors challenged the scarce consideration of PROs in the AIFA’s appraisal: they found that only in 19.7% appraisals published in 2017–2021 were PROs explicitly mentioned in the innovativeness appraisal document. In our analysis this figure increased to 32.9%.

Interestingly, we found a lower likelihood of getting the innovativeness status when the appraisal document cited an ITC (23.5% of cases). This is quite surprising, but since there were no guidelines/recommendations on the requirements for robust evidence, it is likely that ITCs were mentioned when they had not produced convincing results to compensate, at least partially, for the absence of a head-to-head trial.

We observed lower unmet need ratings for the oncologic disease setting, which may reflect the AIFA framework, where the availability of multiple therapeutic alternatives can lead to a lower unmet need classification, even in clinically severe conditions.

This study confirms that the appraisal process is internally consistent. The innovativeness status mostly depends on the added therapeutic value, which is one of the three criteria used to appraise it. A rare indication has a higher likelihood of getting an innovativeness status, consistent with the innovativeness regulation. Other variables play a minor (or not statistically significant) role, with the only exception being the paediatric indications, which could be considered the only systematic deviation from regulation.

This study has some limitations. Data were manually extracted and classified from publicly available AIFA reports, which may have introduced minor errors or interpretative variability despite cross-checking procedures. The original assessment of the three domains inherently involves a degree of subjectivity from AIFA and lacks systematic external validation. The appraisal documents have been enriched over time, making them not fully comparable. Finally, some analyses may have been underpowered due to small sample sizes in specific subgroups, which could have affected confidence intervals and statistical significance.

Despite these limitations the study has provided an up-to-date and complete overview of the Italian experience in the assessment and appraisal of innovativeness, including other variables of interest that have not been considered by the previous empirical studies.

Other HTA authorities have introduced a grading system for the added therapeutic value to support the price or discounts over list prices (and reimbursement) negotiation [11], or broader value frameworks (including the unmet need, the expected added therapeutic value and the necessity/urgency to treat) to allow early access to medicines (e.g., in France [14]). However, the Italian experience is unique in terms of scope (priority access for medicines already approved and reimbursed) and domains (quality of the evidence separately appraised from the added therapeutic value on the grounds of the GRADE system).

## 5. Conclusions

Our study has confirmed that the appraisal of innovativeness in Italy is mainly driven by the added therapeutic value. The process is transparent and internally consistent, too. The innovativeness appraisal documents are published, whereas, on the contrary, only ten complete HTA Reports, including cost-effectiveness analysis, are published on the AIFA’s website [15].

Regardless, the relevant regulation was reformed in July 2025 [10], after a public consultation on a preliminary draft.

The innovativeness criteria were not changed, but (i) conditional approval was abolished, to make the process simpler, considering that in only four cases was the conditional approval converted into full innovativeness; (ii) the companies can apply for innovativeness only if the relevant drug is indicated for a medium–low-prevalence disease, to mitigate the impact on the innovative fund.

More importantly, specific recommendations were included in (i) the criteria to identify the alternative treatments, (ii) the role and robustness of ITCs, (iii) the role and requirements for PROMs.

The first recommendation should make the discussion over the alternative treatments more structured, with less room for discretion: alternative treatments can include the best standard of care used in clinical practice, and reimbursed drugs sharing the same indication or indications that could be considered clinically overlapping. This may have an impact on the predictability of the unmet need and the comparators to consider when assessing and appraising the added therapeutic value.

The second recommendation should make the role of ITCs clearer, their citations in the innovativeness appraisal documents more frequent and their design more consistent with the relevant guidance (anchored comparisons and network meta-analyses are assimilated to RCTs; an unanchored comparison is assimilated to observational studies in the GRADE system). This should invert the evidence collected by our study, i.e., that the citation of ICT is associated with a lower innovativeness status.

Finally, the new regulation has explicitly mentioned that PROMs are an important information source, provided that the data are statistically significant and transferable to the Italian context. This is a formal and important recognition of the role of PROMs and could enhance their role in the evaluation of benefits. Our study has detected an increasing citation of PROMs, but without a statistically significant impact on the appraisal of innovativeness.

## Figures and Tables

**Table 1:** Frequency distribution ^1^ of specific variables according to the final decision on drug innovativeness.

	All Drug Reports (*n* = 255)	Fully Innovative (*n* = 70)	Conditionally Innovative (*n* = 79)	Not Innovative (*n* = 106)	*p*-Value ^2^
Period of Assessment					
2017–2019	67 (26.3)	23 (32.8)	21 (26.6)	23 (21.7)	0.150
2020–2022	119 (46.7)	34 (48.6)	31 (39.2)	54 (50.9)	
2023–2024	69 (27.1)	13 (18.6)	27 (34.2)	29 (27.4)	
Rare Disease					
No	124 (48.6)	30 (42.9)	31 (39.2)	63 (59.4)	0.013
Yes	131 (51.4)	40 (57.1)	48 (60.8)	43 (40.6)	
Orphan Drug Designation					
No	158 (62.0)	35 (50.0)	48 (60.8)	75 (70.7)	0.021
Yes	97 (38.0)	35 (50.0)	31 (39.2)	31 (29.3)	
Type of Disease					
Non-Oncologic	114 (44.7)	32 (45.7)	35 (44.3)	47 (44.3)	0.980
Oncologic	141 (55.3)	38 (54.3)	44 (55.7)	59 (55.7)	
Severity of Disease					
Not Mentioned	131 (51.4)	40 (57.1)	33 (41.8)	58 (54.7)	0.266
Disabling	29 (11.4)	9 (12.9)	10 (12.7)	10 (9.4)	
Lethal or Life-Threatening	95 (37.2)	21 (30.0)	36 (45.6)	38 (35.9)	
Biologic Drug					
No	110 (43.1)	28 (40.0)	34 (43.0)	48 (45.3)	0.787
Yes	145 (56.9)	42 (60.0)	45 (57.0)	58 (54.7)	
Target Population					
Adult-Only	195 (76.5)	42 (60.0)	63 (79.7)	90 (84.9)	<0.001
Paediatric or Mixed	60 (23.5)	28 (40.0)	16 (20.3)	16 (15.1)	
No. of Studies in Support					
1	199 (78.0)	56 (80.0)	62 (78.5)	81 (76.4)	0.848
1+	56 (22.0)	14 (20.0)	17 (21.5)	25 (23.6)	
PROs Mentioned					
No	171 (67.1)	45 (64.3)	48 (60.8)	78 (73.6)	0.157
Yes	84 (32.9)	25 (37.5)	31 (39.2)	28 (26.4)	
ITCs Mentioned					
No	195 (76.5)	62 (88.6)	69 (87.3)	64 (60.3)	<0.001
Yes	60 (23.5)	8 (11.4)	10 (12.7)	42 (39.6)	
Overall Survival					
No	124 (48.6)	33 (47.1)	38 (48.1)	53 (50.0)	0.928
Yes	131 (51.4)	37 (52.9)	41 (51.9)	53 (50.0)	

Abbreviations: ITC: indirect treatment comparison; PROs: patient-reported outcomes. ^1^ Data are expressed as *n* (%) unless otherwise specified. ^2^ From chi-square or Fisher’s exact tests.

**Table 2:** Distribution of evaluations for each of the three domains according to the final decision on drug innovativeness.

	All Drug Reports (*n* = 255)	Fully Innovative (*n* = 70)	Conditionally Innovative (*n* = 79)	Not Innovative (*n* = 106)	*p*-Value ^2^
Unmet Need					
*n*	255	70	79	106	
Maximum (scale = 1)	17 (6.7)	7 (10)	5 (6.3)	5 (4.7)	<0.001 ^2^
Important (scale = 2)	84 (32.9)	34 (48.6)	25 (31.7)	25 (23.5)	
Moderate (scale = 3)	148 (58.0)	29 (41.4)	49 (62.0)	70 (66.0)	
Small (scale = 4)	6 (2.4)	0 (0)	0 (0)	6 (5.7)	
Null (scale = 5)	0 (0.0)	0 (0)	0 (0)	0 (0)	
Mean (SD)	2.56 (0.65)	2.31 (0.65)	2.56 (0.61)	2.73 (0.64)	
Median (25–75°perc)	3 (2–3)	2 (2–3)	3 (2–3)	3 (2–3)	<0.001 ^3^
Added Therapeutic Value ^1^					
*n*	234	70	79	85	
Maximum (scale = 1)	2 (0.9)	1 (1.4)	1 (1.3)	0 (0)	<0.001 ^2^
Important (scale = 2)	58 (24.8)	54 (77.1)	1 (1.3)	3 (3.5)	
Moderate (scale = 3)	114 (48.7)	15 (21.4)	77 (97.5)	22 (25.9)	
Small (scale = 4)	47 (20.1)	0 (0)	0 (0)	47 (55.3)	
Null (scale = 5)	13 (5.6)	0 (0)	0 (0)	13 (15.3)	
Mean (SD)	3.05 (0.84)	2.2 (0.44)	2.96 (0.25)	3.82 (0.73)	
Median (25–75°perc)	3 (2–4)	2 (2–2)	3 (3–3)	4 (3–4)	<0.001 ^3^
GRADE					
*n*	255	70	79	106	
High (scale = 1)	34 (13.3)	17 (24.3)	9 (11.4)	8 (7.6)	<0.001 ^2^
Moderate (scale = 2)	119 (46.7)	34 (48.6)	43 (54.4)	42 (39.6)	
Low (scale = 3)	72 (28.2)	14 (20)	25 (31.7)	33 (31.1)	
Very low (scale = 4)	30 (11.8)	5 (7.1)	2 (2.5)	23 (21.7)	
Mean (SD)	2.38 (0.86)	2.1 (0.85)	2.25 (0.69)	2.67 (0.9)	
Median (25–75°perc)	2 (2–3)	2 (2–3)	2 (2–3)	3 (2–3)	<0.001 ^3^

Abbreviations: GRADE: Grading of Recommendations, Assessment, Development, and Evaluation; perc: percentiles; SD: standard deviation. ^1^ In a few cases (*n* = 21), the added therapeutic value was deemed ‘not evaluable’. ^2^ From Fisher’s exact tests (domain evaluation as a categorical variable). ^3^ From Kruskal–Wallis tests (domain evaluation as quantitative ordinal variable).

**Table 3:** Multiple adjusted odds ratios (ORs) and 95% confidence intervals (CIs) for the association between selected factors and the final decision on drug innovativeness.

	Fully Innovative vs. Not Innovative (OR ^1^; 95 CI)	Conditionally Innovative vs. Not Innovative (OR ^1^; 95 CI)	Innovative (Fully or Conditionally) vs. Not Innovative (OR ^2^; 95 CI)
Period of assessment			
2017–2019	1 (reference)	1 (reference)	1 (reference)
2020–2022	0.40 (0.17–0.93)	0.42 (0.18–0.99)	0.42 (0.20–0.86)
2023–2024	0.32 (0.11–0.89)	0.70 (0.28–1.77)	0.51 (0.22–1.17)
Rare disease			
No	1 (reference)	1 (reference)	1 (reference)
Yes	2.20 (1.06–4.58)	2.95 (1.48–5.92)	2.59 (1.41–4.78)
Severity of the disease			
Not mentioned	1 (reference)	1 (reference)	1 (reference)
Disabling	0.57 (0.18–1.83)	1.01 (0.33–3.15)	0.77 (0.29–2.10)
Lethal or life-threatening	0.80 (0.37–1.71)	1.38 (0.68–2.79)	1.07 (0.58–2.00)
Target population			
Adult only	1 (reference)	1 (reference)	1 (reference)
Paediatric or mixed	3.60 (1.53–8.44)	1.18 (0.49–2.86)	2.19 (0.98–4.45)
PROs mention			
No	1 (reference)	1 (reference)	1 (reference)
Yes	1.72 (0.79–3.75)	1.68 (0.81–3.49)	1.69 (0.88–3.23)
ITCs mention			
No	1 (reference)	1 (reference)	1 (reference)
Yes	0.18 (0.08–0.45)	0.18 (0.08–0.41)	0.18 (0.09–0.36)
Domains of innovativeness			
Unmet need ^3^	1.89 (1.09–3.29)	1.34 (0.77–2.33)	1.59 (0.98–2.59)
Added therapeutic value ^3^	∞	47.8 (11.53–198.63)	70.18 (17.64–279.26)
GRADE ^3^	3.79 (2.25–6.41)	2.12 (1.35–3.35)	2.67 (1.77–4.03)

Abbreviations: GRADE: Grading of Recommendations, Assessment, Development, and Evaluation; ITCs: indirect treatment comparisons; PROs: patient-reported outcomes. ^1^ Derived from multinomial logistic regression models, including simultaneously the factors in the table and, in turn, the three domains of unmet need, added therapeutic value, and quality of clinical evidence (GRADE). The ORs for orphan drug designation (derived from the same model but excluding the rarity of the disease) were 2.50 (95% CI: 1.18–5.31) for fully and 1.87 (95% CI: 0.92–3.82) for partially innovative vs. not innovative. ^2^ Derived from logistic regression models, including simultaneously the factors in the table and, in turn, the three domains of unmet need, added therapeutic value, and quality of clinical evidence (GRADE evaluation). The OR for orphan drug designation (derived from the same model but excluding the rarity of the disease) was 2.12 (95% CI: 1.12–4.01). ^3^ Continuous OR, for an improvement of 1 level in the scale of evaluation.

## Data Availability

The data underlying this study are derived from publicly available AIFA reports. The extracted dataset is available from the corresponding author upon reasonable request.

## Supplementary Materials

The following supporting information can be downloaded at https://www.mdpi.com/article/10.3390/jmahp14020028/s1. Table S1: Crude odds ratios (ORs) and 95% confidence intervals (CIs) for the association between selected factors and the final decision on drug innovativeness; Table S2: Association between selected factors and evaluation in the unmet need domain; Table S3: Association between selected factors and evaluation in the added therapeutic value domain; Table S4: Association between selected factors and evaluation in the GRADE domain. File S1. Dataset structure, variables, and data extraction process.

## Abbreviations

The following abbreviations are used in this manuscript:

AIFA	Italian Medicines Agency
OR	Odds Ratio
CI	Confidence Interval
P&R	Price and Reimbursement
GRADE	Grading of Recommendations Assessment, Development and Evaluation
ITC	Indirect Treatment Comparison
PROM	Patient-Reported Outcome Measure
PREM	Patient-Reported Experience Measure
HTA	Health Technology Assessment
