# Supplementary material for: Assessment and Appraisal of Drug Innovativeness in Italy: Ultimate Evidence on Key Drivers and Consistency
Source: J Mark Access Health Policy. 2026 May 2;14(2):28. doi: 10.3390/jmahp14020028 (PMC13214850; doi:10.3390/jmahp14020028)
Supplement: Supplementary file 1 [file jmahp-14-00028-s001.zip › jmahp-4186625-supplementary.pdf]

Supplementary Information

Assessment and Appraisal of Drug Innovativeness in Italy: the ultimate evidence on key drivers and consistency

Journal of Market Access and Health Policy

Alvise Verde, Federica Turati, Clara Trimarchi, Carlotta Galeone, Claudio Jommi*

* Department of Pharmaceutical Sciences, Università del Piemonte Orientale, Novara, Italy; claudio.jommi@uniupo.it.

File S1. Dataset structure, variables, and data extraction process

The dataset includes 255 entries, each corresponding to a single innovativeness appraisal per therapeutic indication issued by the Italian Medicines Agency (AIFA) between July 2017 and December 2024.

Each entry derives from an official AIFA innovativeness appraisal document published after the conclusion of the price and reimbursement (P&R) procedure. Data were manually extracted from publicly available reports and organized into a structured Excel database.

Primary data source: public AIFA innovativeness appraisal documents.
https://www.aifa.gov.it/farmaci-innovativi

Variables included in the dataset

Table SA: Identification Variables

Variable name	Description	Type	Units / Values
Trade name	Commercial name of the medicine	Categorical	Text
Active substance	Active substance or combination	Categorical	Text
Indication	Therapeutic indication under appraisal	Categorical	Text
Year of appraisal	Year of publication of the P&R decision	Discrete	Calendar year

Table SB: Dependent Variable

Variable name	Description	Type	Values
Innovativeness status	Final innovativeness appraisal outcome	Nominal categorical	non-innovative / conditional / full

Table SC: AIFA's Innovativeness Domains

Variable name	Description	Type	Scale
Unmet need	Evaluation of unmet therapeutic need	Ordinal	maximum / important / moderate / small / null
Added therapeutic value	Evaluation of added therapeutic value	Ordinal	maximum / important / moderate / small / null
Quality of Evidence	Quality of clinical evidence according to GRADE	Ordinal	high / moderate / low / very low

The rating scales are consistent with AIFA's innovativeness framework and the GRADE methodology, as explicitly described in the manuscript.

Table SD: Disease- and Drug-Related Characteristics

Variable name	Description	Type	Values
Rare disease	Rare disease indication	Binary	yes / no
Orphan designation	Orphan drug designation	Binary	yes / no
Disease type	Therapeutic area	Binary	oncologic / non-oncologic
Disease severity	Disease severity as reported in the appraisal document	Categorical	not mentioned / disabling / lethal or life-threatening
Biologic drug	Biologic nature of the medicine	Binary	yes / no

Table SE: Target Population

Variable name	Description	Type	Values
Target population	Target population of the indication	Binary	adult only / paediatric or mixed

Table SF: Clinical and Methodological Evidence

Variable name	Description	Type	Values
Number of studies	Number of clinical studies considered	Binary	1 / >1
PROs mentioned	Explicit mention of patient-reported outcomes (PROs)	Binary	yes / no
ITCs mentioned	Explicit mention of indirect treatment comparison (ITC)	Binary	yes / no
Overall survival	Overall survival evaluated as clinical endpoint	Binary	yes / no

The variables related to PROs and ITCs capture only their explicit mention in the AIFA appraisal documents and do not reflect their quality, statistical significance, or impact.

Missing data

In a limited number of cases:

- the added therapeutic value was reported as *not evaluable*.
- disease severity was not explicitly mentioned in the appraisal document.

These cases were retained as explicit categories or missing values, without data imputation.

Data collection process

- Manual extraction from public AIFA appraisal documents.
- Cross-checking during data coding to ensure internal consistency.
- No automated data transformations or imputations applied.

Table S1. Crude odds ratios (OR) and 95% confidence intervals (CI) for the association between selected factors and the final decision on drug innovativeness.

	Full Innovative vs. Not Innovative (OR[a]; 95 CI)	Conditional Innovative vs Not Innovative (OR[a]; 95 CI)	Innovative (full or Conditional) vs Not Innovative (OR[b]; 95 CI)
Period of Assessment			
2017-2019	1 (reference)	1 (reference)	1 (reference)
2020-2022	0.63 (0.31-1.29)	0.63 (0.30-1.32)	0.63 (0.34-1.17)
2023-2024	0.45 (0.19-1.07)	1.02 (0.46-2.25)	0.72 (0.36-1.44)
Rare Disease			
No	1 (reference)	1 (reference)	1 (reference)
Yes	1.95 (1.06-3.60)	2.27 (1.25-4.11)	2.11 (1.27-3.51)
Orphan Drug Designation			
No	1 (reference)	1 (reference)	1 (reference)
Yes	2.42 (1.29-4.54)	1.56 (0.84-2.89)	1.92 (1.13-3.26)
Type of disease			
Non-oncologic	1 (reference)	1 (reference)	1 (reference)
Oncologic	0.95 (0.52-1.74)	1.00 (0.56-1.80)	0.98 (0.59-1.61)
Severity of the disease			
Not mentioned	1 (reference)	1 (reference)	1 (reference)
Disabling	1.31 (0.49-3.50)	1.76 (0.66-4.66)	1.51 (0.65-3.50)
Lethal or life-threatening	0.80 (0.41-1.56)	1.665 (0.891-3.11)	1.19 (0.70-2.04)
Biologic drug			
No	1 (reference)	1 (reference)	1 (reference)
Yes	1.24 (0.67-2.29)	1.10 (0.61-1.97)	1.16 (0.70-1.92)
Target population			
Adult only	1 (reference)	1 (reference)	1 (reference)
Paediatric or mixed	3.75 (1.83-7.67)	1.43 (0.67-3.07)	2.36 (1.25-4.46)
Total n. of studies in support			
1	1 (reference)	1 (reference)	1 (reference)
>1	0.81 (0.39-1.69)	0.89 (0.44-1.79)	0.85 (0.47-1.55)
PROs mention			
No	1 (reference)	1 (reference)	1 (reference)
Yes	1.55 (0.81-2.97)	1.80 (0.96-3.36)	1.68 (0.97-2.89)
ITCs mention			
No	1 (reference)	1 (reference)	1 (reference)
Yes	0.20 (0.09-0.45)	0.22 (0.10-0.48)	0.21 (0.11-0.39)
Overall survival			
No	1 (reference)	1 (reference)	1 (reference)
Yes	1.12 (0.61-2.05)	1.08 (0.60-1.93)	1.10 (0.67-1.81)
Domains of innovativeness			
Unmet need	2.71 (1.65-4.44)	1.59 (0.98-2.58)	2.05 (1.34-3.12)
Added therapeutic value	∞	38.6 (11.3-131.86)	53.5 (16.17-177.26)
GRADE evaluation	2.30 (1.55-3.41)	1.80 (1.26-2.59)	2.01 (1.47-2.76)

Abbreviations: GRADE: Grading of Recommendations, Assessment, Development, and Evaluation; ITCs: indirect treatment comparisons; PROs: patient reported outcomes.

[a] Derived from unadjusted multinomial logistic regression models.

[b] Derived from unadjusted logistic regression models.

Table S2. Association between selected factors and evaluation in the unmet need domain (frequencies, p-values, and ordinal logistic regression results)[a].

	Frequency (n, %)				p-value[b]	Crude OR[c] (95% CI)	Adjusted OR[d] (95% CI) (main results)
	Maximum (n=17)	Important (n=84)	Moderate (n=148)	Small (n=6)			
Period of Assessment							
2017-2019	8 (47.1)	30 (35.7)	24 (16.2)	5 (83.3)	*<0.001*	1 (reference)	1 (reference)
2020-2022	7 (41.2)	36 (42.9)	76 (51.4)	0 (0.0)		0.51 (0.29-0.92)	0.39 (0.20-0.75)
2023-2019	2 (11.8)	18 (21.4)	48 (32.4)	1 (16.7)		0.36 (0.18-0.71)	0.33 (0.15-0.70)
Rare Disease							
No	3 (17.7)	43 (51.2)	75 (50.7)	3 (50.0)	*0.063*	1 (reference)	1 (reference)
Yes	14 (82.5)	41 (48.8)	73 (49.3)	3 (50.0)		1.36 (0.83-2.21)	1.41 (0.81-2.44)
Orphan Drug Designation[e]							
No	5 (29.4)	48 (57.1)	101 (68.2)	4 (66.7)	*0.011*	1 (reference)	-
Yes	12 (70.6)	36 (42.9)	47 (31.8)	2 (33.3)		2.07 (1.25-3.41)	
Type of disease							
Non-oncologic	12 (70.6)	48 (57.1)	50 (33.8)	4 (66.7)	*<0.001*	1 (reference)	1 (reference)
Oncologic	5 (29.4)	36 (42.9)	98 (66.2)	2 (33.3)		0.40 (0.24-0.66)	0.50 (0.27-0.93)
Severity of the disease							
Not mentioned	7 (41.2)	51 (60.7)	67 (45.3)	6 (100)	*0.015*	1 (reference)	1 (reference)
Disabling	2 (11.8)	12 (14.3)	15 (10.1)	0 (0.0)		1.30 (0.59-2.80)	0.64 (0.26-1.57)
Lethal or life-threatening	8 (47.1)	21 (25.0)	66 (44.6)	0 (0.0)		0.71 (0.42-1.21)	0.85 (0.47-1.52)
Biologic drug							
No	4 (23.5)	43 (51.2)	59 (39.9)	4 (66.7)	*0.072*	1 (reference)	1 (reference)
Yes	13 (76.5)	41 (48.8)	89 (60.1)	2 (33.3)		0.94 (0.58-1.54)	1.12 (0.66-1.89)
Target population							
Adult only	6 (35.3)	61 (72.6)	124 (83.8)	4 (66.7)	*<0.001*	1 (reference)	1 (reference)
Paediatric or mixed	11 (64.7)	23 (27.4)	24 (16.2)	2 (33.3)		2.82 (1.60-4.96)	2.53 (1.31-4.89)
Total n. of studies in support							
1	10 (58.8)	61 (72.6)	124 (83.8)	4 (66.7)	*0.026*	1 (reference)	1 (reference)
>1	7 (41.2)	23 (27.4)	24 (16.2)	2 (33.3)		2.05 (1.15-3.64)	1.26 (0.66-2.41)
PROs mention							
No	14 (82.4)	57 (67.9)	95 (64.2)	5 (83.3)	*0.450*	1 (reference)	1 (reference)
Yes	3 (17.6)	27 (32.1)	53 (35.8)	1 (1667)		0.79 (0.47-1.33)	0.83 (0.46-1.50)
ITC							
No	16 (94.1)	71 (84.5)	104 (70.3)	4 (66.7)	*0.017*	1 (reference)	1 (reference)
Yes	1 (5.9)	13 (15.5)	44 (29.7)	2 (33.3)		0.39 (0.21-0.73)	0.40 (0.20-0.77)
Overall survival[f]							
No	12 (70.6)	53 (63.1)	55 (37.2)	4 (66.7)		1 (reference)	-
Yes	5 (29.4)	31 (36.9)	93 (62.8)	2 (33.3)	<0.001	0.38 (0.23-0.63)	

Abbreviations: CI, confidence interval; ITCs: indirect treatment comparisons; OR: odds ratio; PROs: patient reported outcomes.

[a] Percentages are reported by column. P-values refer to overall comparisons across categories. Ordinal logistic regression models estimate the association between each factor and higher ratings.

[b] From Chi-square or Fisher exact test.

[c] Derived from an ordered logistic regression model.

[d] Derived from an ordered logistic regression model, including simultaneous the factors in Table with the exception of orphan drug designation (highly associated with the rarity of disease) and overall survival (highly associated with the oncologic disease type).

[e] The adjusted OR for orphan drug designation (derived from the same model but excluding the rarity of the disease) was 2.15 (95% CI: 1.22-3.80).

[f] The adjusted OR for overall survival (derived from the same model but excluding the type of disease) was 0.52 (95% CI: 0.29-0.94).

Table S3. Association between selected factors and evaluation in the added therapeutic value domain (frequencies, p-values, and ordinal logistic regression results)[a].

	Frequency (n, %)					p-value[b]	Crude OR[c] (95% CI)	Adjusted OR[d] (95% CI) (main results)
	Max (n=2)	Import. (n=58)	Mod. (n=114)	Small (n=47)	Null (n=13)			
Period of Assessment								
2017-2019	1 (50)	20 (34.5)	28 (24.6)	16 (34.0)	1 (7.7)	*0.079*	1 (reference)	1 (reference)
2020-2022	1 (50)	28 (48.3)	48 (42.1)	23 (48.9)	7 (53.9)		0.79 (0.45-1.41)	0.73 (0.39-1.39)
2023-2019	0 (0)	10 (17.2)	38 (33.3)	8 (17.0)	5 (38.5)		0.70 (0.37-1.34)	0.66 (0.31-1.38)
Rare Disease								
No	1 (50)	23 (39.7)	52 (45.6)	29 (61.7)	4 (30.8)	*0.082*	1 (reference)	1 (reference)
Yes	1 (50)	35 (60.3)	62 (54.4)	18 (38.3)	9 (69.2)		1.42 (0.88-2.30)	1.44 (0.84-2.45)
Orphan Drug Designation[e]								
No	2 (100)	27 (46.6)	69 (60.5)	33 (70.2)	9 (69.2)	*0.116*	1 (reference)	-
Yes	0 (0)	31 (53.5)	45 (39.5)	14 (29.8)	4 (30.8)		1.80 (1.10-2.96)	
Type of disease								
Non-oncologic	1 (50)	24 (41.4)	55 (48.3)	26 (55.3)	5 (38.5)	*0.490*	1 (reference)	1 (reference)
Oncologic	1 (50)	34 (58.6)	59 (51.8)	21 (44.7)	8 (61.5)		1.27 (0.79-2.05)	1.58 (0.87-2.88)
Severity of the disease								
Not mentioned	1 (50)	36 (62.1)	48 (42.1)	27 (57.5)	7 (53.9)	*0.095*	1 (reference)	1 (reference)
Disabling	0 (0)	3 (5.2)	17 (14.9)	8 (17.0)	1 (7.7)		0.59 (0.28-1.25)	0.41 (0.17-0.97)
Lethal or life-threatening	1 (50)	19 (32.8)	49 (43.0)	12 (25.5)	5 (38.5)		1.01 (0.60-1.69)	0.90 (0.51-1.57)
Biologic drug								
No	1 (50)	24 (41.4)	48 (42.1)	21 (44.7)	7 (53.9)	*0.860*	1 (reference)	1 (reference)
Yes	1 (50)	34 (58.6)	66 (57.9)	26 (55.3)	6 (46.1)		1.16 (0.72-1.88)	1.16 (0.69-1.93)
Target population								
Adult only	1 (50)	38 (65.5)	86 (75.4)	39 (83.0)	12 (92.3)	*0.075*	1 (reference)	1 (reference)
Paediatric or mixed	1 (50)	20 (34.5)	28 (24.6)	8 (17.0)	1 (7.7)		2.11 (1.20-3.71)	2.81 (1.45-5.43)
Total n. of studies in support								
1	2 (100)	46 (79.3)	89 (78.1)	33 (70.2)	11 (84.6)	*0.566*	1 (reference)	1 (reference)
>1	0 (0)	12 (20.7)	25 (21.9)	14 (29.8)	2 (15.4)		0.80 (0.45-1.41)	0.85 (0.45-1.62)
PROs mention								
No	2 (100)	40 (69.0)	72 (63.2)	30 (63.8)	10 (76.9)	*0.655*	1 (reference)	1 (reference)
Yes	0 (0)	18 (31.0)	42 (36.8)	17 (36.2)	3 (23.1)		0.92 (0.56-1.52)	0.91 (0.52-1.60)
ITC								
No	2 (100)	51 (87.9)	93 (81.6)	26 (55.3)	6 (46.1)	*<0.001*	1 (reference)	1 (reference)
Yes	0 (0)	7 (12.0)	21 (18.4)	21 (44.7)	7 (53.9)		0.25 (0.14-0.45)	0.24 (0.13-0.44)
Overall survival[f]								
No	1 (50)	24 (41.4)	59 (51.8)	29 (61.7)	5 (38.5)	*0.168*	1 (reference)	-
Yes	1 (50)	34 (58.6)	55 (48.3)	18 (38.3)	8 (61.5)		1.44 (0.89-2.33)	

Abbreviations: CI, confidence interval; Import.: important; ITCs: indirect treatment comparisons; Mod.: moderate; OR: odds ratio; PROs: patient reported outcomes.

[a] Percentages are reported by column. P-values refer to overall comparisons across categories. Ordinal logistic regression models estimate the association between each factor and higher ratings.

[b] From Chi-square or Fisher exact test. The test was conducted after combining the "maximum" and "important" categories of the added therapeutic value variable.

[c] Derived from an ordered logistic regression model.

[d] Derived from an ordered logistic regression model, including simultaneous the factors in Table with the exception of orphan drug designation (highly associated with the rarity of disease) and overall survival (highly associated with the oncologic disease type).

[e] The adjusted OR for orphan drug designation (derived from the same model but excluding the rarity of the disease) 2.01 (95% CI: 1.17-3.48).

[f] The adjusted OR for overall survival (derived from the same model but excluding the type of disease) was 1.83 (95% CI: 1.02-3.30).

Table S4. Association between selected factors and evaluation in the GRADE evaluation domain (frequencies, p-values, and ordinal logistic regression results)[a].

	Frequency (n, %)				p-value[b]	Crude OR[c] (95% CI)	Adjusted OR[d] (95% CI) (main results)
	High (n=34)	Moderate (n=119)	Low (n=72)	Very low (n=30)			
Period of Assessment							
2017-2019	10 (29.4)	37 (31.1)	14 (19.4)	6 (20.0)	*0.587*	1 (reference)	1 (reference)
2020-2022	15 (44.1)	51 (42.9)	36 (50.0)	17 (56.7)		0.61 (0.35-1.06)	0.49 (0.27-0.92)
2023-2019	9 (26.5)	31 (26.0)	22 (30.6)	7 (23.3)		0.69 (0.37-1.30)	0.54 (0.26-1.09)
Rare Disease							
No	26 (76.5)	60 (50.4)	31 (43.1)	7 (23.3)	*<0.001*	1 (reference)	1 (reference)
Yes	8 (23.5)	59 (49.6)	41 (56.9)	23 (76.7)		0.38 (0.24-0.61)	0.42 (0.25-0.71)
Orphan Drug Designation[e]							
No	27 (79.4)	79 (66.4)	40 (55.6)	12 (40.0)	*0.005*	1 (reference)	-
Yes	7 (20.6)	40 (33.6)	32 (44.4)	18 (60.0)		0.43 (0.27-0.69)	
Type of disease							
Non-oncologic	10 (29.4)	47 (39.5)	42 (58.3)	15 (50.0)	*0.016*	1 (reference)	1 (reference)
Oncologic	24 (70.6)	72 (60.5)	30 (41.7)	15 (50.0)		1.97 (1.24-3.14)	1.64 (0.93-2.90)
Severity of the disease							
Not mentioned	20 (58.8)	60 (50.4)	37 (51.4)	14 (46.7)	*0.859*	1 (reference)	1 (reference)
Disabling	2 (5.9)	15 (12.6)	7 (9.7)	5 (16.7)		0.72 (0.34-1.52)	1.63 (0.69-3.84)
Lethal or life-threatening	12 (35.2)	44 (37.0)	28 (38.9)	11 (36.7)		0.89 (0.54-1.45)	0.99 (0.58-1.69)
Biologic drug							
No	17 (50.0)	50 (42.0)	34 (47.2)	9 (30.0)	*0.346*	1 (reference)	1 (reference)
Yes	17 (50.0)	69 (58.0)	38 (52.8)	21 (70.0)		0.82 (0.51-1.30)	0.83 (0.51-1.35)
Target population							
Adult only	33 (97.1)	95 (79.8)	49 (68.1)	18 (6.0)	*0.001*	1 (reference)	1 (reference)
Paediatric or mixed	1 (2.9)	24 (20.2)	23 (31.9)	12 (40.0)		0.34 (0.20-0.60)	0.46 (0.25-0.86)
Total n. of studies in support							
1	29 (85.3)	101 (84.9)	48 (66.7)	21 (70.0)	*0.013*	1 (reference)	1 (reference)
>1	5 (14.7)	18 (15.1)	24 (33.3)	9 (30.0)		0.44 (0.25-0.77)	0.53 (0.29-0.99)
PROs mention							
No	19 (55.9)	75 (63.0)	51 (70.8)	26 (86.7)	*0.037*	1 (reference)	1 (reference)
Yes	15 (44.1)	44 (37.6)	21 (29.2)	4 (13.3)		1.98 (1.20-3.24)	2.46 (1.42-4.26)
ITC							
No	30 (88.2)	93 (78.2)	58 (80.6)	14 (46.7)	*<0.001*	1 (reference)	1 (reference)
Yes	4 (11.8)	26 (21.8)	14 (19.4)	16 (53.3)		0.44 (0.26-0.75)	0.41 (0.23-0.74)
Overall survival[f]							
No	11 (32.4)	53 (44.54)	43 (59.7)	17 (56.7)	*0.033*	1 (reference)	-
Yes	23 (67.6)	66 (55.46)	29 (40.3)	13 (43.3)		1.94 (1.22-3.08)	

Abbreviations: CI, confidence interval; GRADE: Grading of Recommendations, Assessment, Development, and Evaluation; ITCs: indirect treatment comparisons; OR, odds ratio; PROs: patient reported outcomes.

[a] Percentages are reported by column. P-values refer to overall comparisons across categories. Ordinal logistic regression models estimate the association between each factor and higher ratings.

[b] From Chi-square or Fisher exact test.

[c] Derived from an ordered logistic regression model.

[d] Derived from an ordered logistic regression model, including simultaneous the factors in Table with the exception of orphan drug designation (highly associated with the rarity of disease) and overall survival (highly associated with the oncologic disease type).

[e] The adjusted OR for orphan drug designation (derived from the same model but excluding the rarity of the disease) was 0.50 (95% CI: 0.30-0.85).

[f] The adjusted OR for overall survival (derived from the same model but excluding the type of disease) was 1.86 (95% CI: 1.07-3.24).
